# Cytomegalovirus and Herpes Simplex Virus Co-Infection in an HIV-Negative Patient: A Case Report

**DOI:** 10.7759/cureus.13214

**Published:** 2021-02-08

**Authors:** Angelica C Gangemi, Sung H Choi, Zhiwei Yin, Mirela Feurdean

**Affiliations:** 1 Internal Medicine-Pediatrics, Christiana Care, Nemours Alfred I. duPont Hospital for Children, Newark, USA; 2 Internal Medicine, Rutgers New Jersey Medical School, Newark, USA; 3 Pathology, Memorial Sloan Kettering Cancer Center, New York, USA

**Keywords:** cytomegalovirus (cmv), hsv-2, cell-mediated immunity, odynophagia, cytomegalovirus esophagitis, cytomegalovirus pneumonitis, herpes simplex virus, hsv-1, oral ulcer, immunosenescence

## Abstract

Herpes simplex virus (HSV) and cytomegalovirus (CMV) infections are commonly seen in immunocompromised patients, particularly in patients with HIV. However, fulminant CMV infection and concurrent infection with HSV and CMV in non-HIV patients are quite rare. We present the case of a 72-year-old HIV-negative man with a history of oropharyngeal carcinoma in remission and recent treatment of immune thrombocytopenic purpura with high-dose steroids who was transferred from an outside hospital for Ear Nose and Throat (ENT) evaluation of a non-healing buccal ulcer. During initial presentation, the patient was found to be febrile with acute hypoxic respiratory failure and a chest x-ray suggestive of bacterial pneumonia, though he failed to improve with broad-spectrum antibiotic therapy. He underwent esophagogastroduodenoscopy for dysphagia, which revealed a discrete ulcer positive for CMV. Biopsy of his buccal lesion was ultimately positive for HSV-1 and HSV-2. The patient’s clinical status improved significantly following the initiation of antiviral therapy.It is important to consider CMV infection in the setting of persistent fever, respiratory distress, or dysphagia in the non-HIV infected patient, especially in the setting of prolonged steroid use. CMV and HSV infection can occur simultaneously at distinct sites in the body, and CMV infection may predispose to HSV reactivation due to its long term effect on cell-mediated immunity. Early recognition of opportunistic infections and initiation of antiviral therapy in immunocompromised patients can greatly affect length of hospital stay, morbidity, and, ultimately, mortality.

## Introduction

Herpes simplex virus (HSV) and cytomegalovirus (CMV) infections are often seen in immunocompromised patients, particularly in patients with HIV [1]. However, fulminant CMV infection is not often seen in immunocompromised patients without HIV [2]. Furthermore, co-infection with HSV and CMV in non-HIV patients is quite rare. To date, there are only two published case reports of concurrent HSV and CMV infections in patients without HIV [3,4].

Early recognition of opportunistic infections in immunocompromised patients can affect length of hospital stay, morbidity, and, ultimately, mortality. This report describes the case of a patient who presented with a non-healing buccal ulcer and was found to be in acute respiratory distress due to suspected bacterial pneumonia. The patient did not respond to broad-spectrum antibiotics and his clinical condition rapidly worsened. However, recognition and identification of concurrent disseminated cytomegalovirus and herpes simplex infections led to early intervention with antiviral therapy, resulting in rapid improvement and stabilization of the patient’s respiratory status.

## Case presentation

A 72-year-old man with a history of squamous cell carcinoma of the oropharynx (treated 10 years prior with radical resection of the oropharyngeal lesion, chemotherapy, and local radiation) and recent hospital admission for immune thrombocytopenic purpura (treated with high-dose steroids) presented from an outside hospital for Ear Nose and Throat (ENT) evaluation of a non-healing buccal ulcer present for three weeks. He also reported worsening odynophagia and 20-pound weight loss over two months that prompted his family to bring him to the outside hospital for evaluation. At the outside hospital, the patient was hypoxic, with oxygen saturation in the high 80s in room air, and a chest x-ray revealed a right lower lung opacity, suggestive of pneumonia. He was started on empiric antibiotic therapy with vancomycin and piperacillin/tazobactam for presumed healthcare-associated pneumonia. Given his past medical history and reported weight loss, the buccal lesion was suspicious of recurrent malignancy, and he was transferred to our hospital for further workup. 

**Figure 1:**
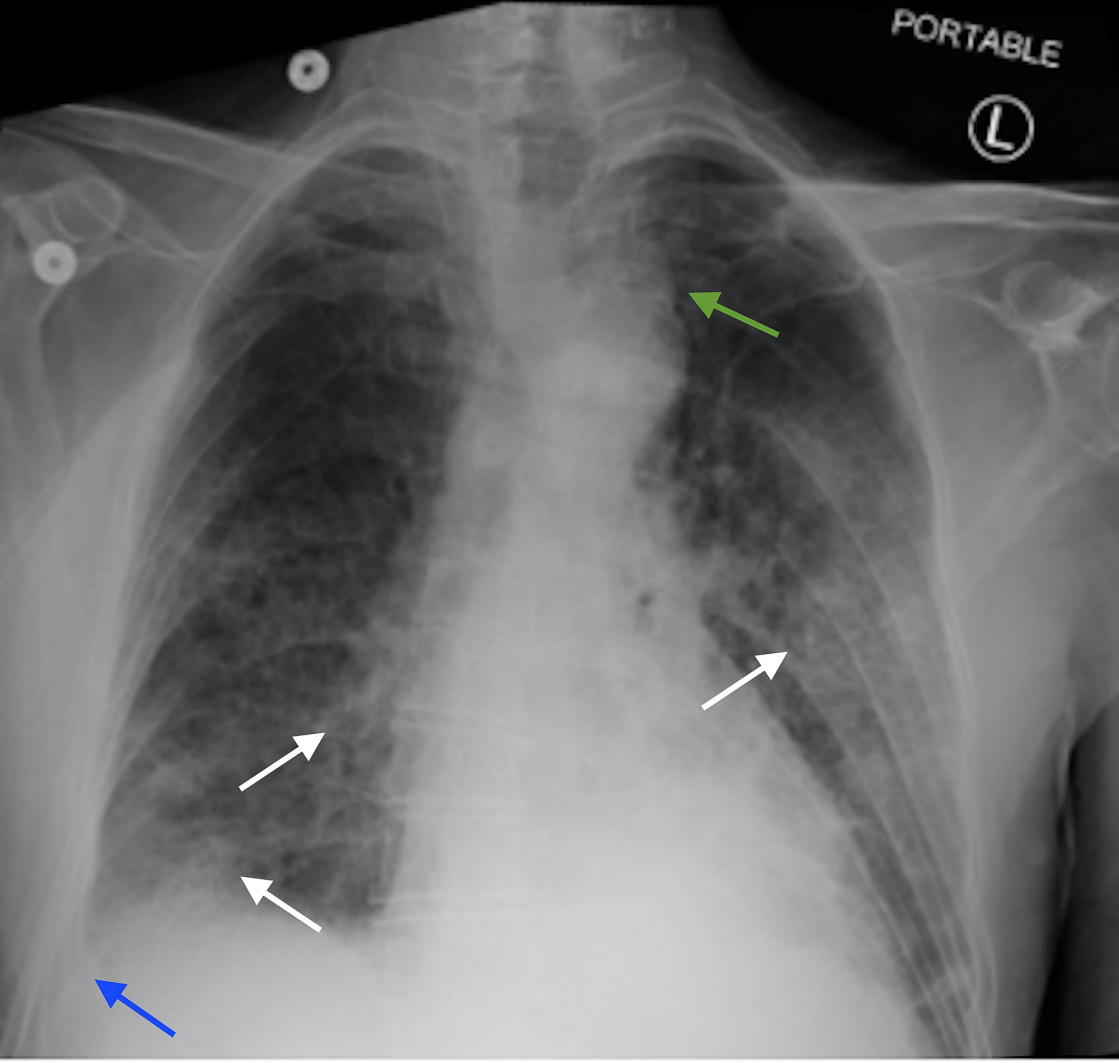
Admission Chest X-ray Initial chest X-ray demonstrating left perihilar, right mid and lower lung patchy opacities, which may represent pneumonia or aspiration (white arrows). There is blunting of the right costophrenic angle may represent a small pleural effusion or pleural thickening (blue arrow). There is a left paratracheal opacity, superior to the aortic arch, which may represent lymphadenopathy (green arrow).

At the time of transfer, the patient was afebrile (98.8 degrees Fahrenheit) with a blood pressure of 108/64 mmHg, heart rate of 82, respiratory rate of 18, and oxygen saturation of 100% on non-rebreather face mask at 80% fraction of inspired oxygen (FiO2). Physical examination revealed a cachectic man with oral thrush, a small area of erythema with central ulceration on the posterior left buccal mucosa and diminished lung sounds on the right. Labs demonstrated a white blood cell count of 9.5 x 10^3/µl, hemoglobin of 11.2 g/dL, and platelets of 72 x 10^3/µl (baseline unknown). Chest x-ray demonstrated right middle and lower lobe opacities, suggestive of hospital-acquired pneumonia versus aspiration pneumonia given the odynophagia, or metastatic disease from recurrent oral cancer (Figure 1). Vancomycin and piperacillin/tazobactam were continued and nystatin was started to treat the oral thrush. A modified barium swallow examination performed under fluoroscopic guidance determined the patient had severe dysphagia and recommended that the patient receive nothing by mouth. Gastroenterology was consulted for placement of a percutaneous endoscopic gastrostomy (PEG) tube. ENT planned to biopsy the left buccal mucosa to confirm the suspicion of recurrent oral cancer. 

**Figure 2:**
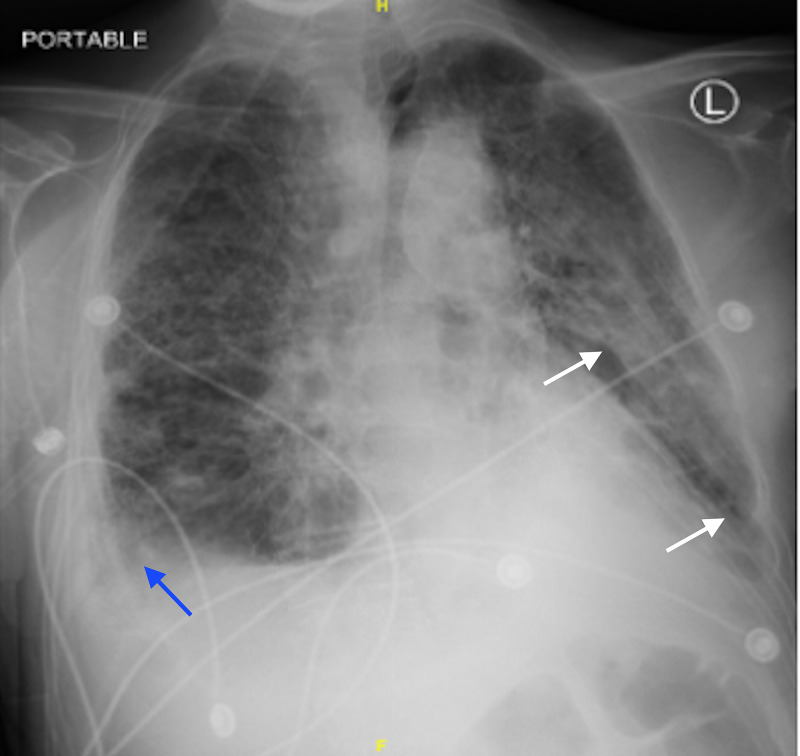
Repeat Chest X-ray on Antibiotic Therapy Chest X-Ray taken on day six of antibiotics, prior to initiation of antiviral therapy. A right basilar opacity is compatible with a layering pleural effusion (blue arrow). Left perihilar and left basilar consolidation is also noted (white arrows).

Over the next few days of admission, the patient developed low-grade fevers and acute hypoxic respiratory failure with multiple episodes of desaturation to the high 80s despite being on 3L nasal cannula. He ultimately required high flow oxygen and subsequent bilevel positive airway pressure machine (BiPAP). Repeat chest x-ray showed increased bilateral lung opacities suggestive of pleural effusions despite broad-spectrum antibiotic therapy (Figure 2).

**Figure 3:**
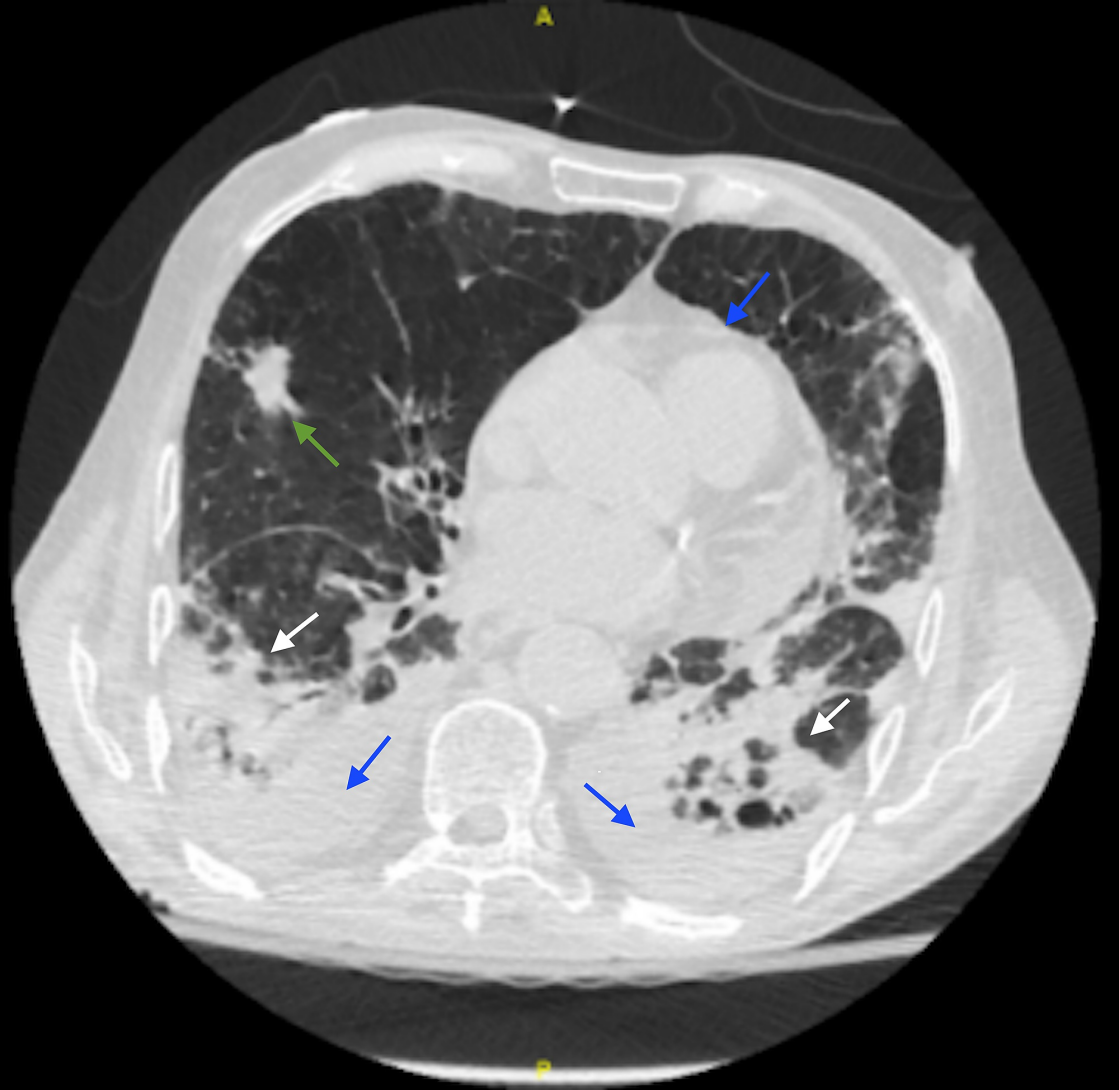
Chest CT on Day of Antiviral Initiation CT Chest taken on day nine of antibiotics and the day of antiviral initiation. Bilateral upper and lower lobe consolidations compatible with multifocal pneumonia (white arrow). Moderate-sized pleural effusions and moderate-sized pericardial effusion is also noted (blue arrows). There is a spiculated nodule in the right middle lobe, concerning for neoplasm (green arrow).

Given the patient’s worsening respiratory status, piperacillin/tazobactam was discontinued and antibiotics were broadened to include meropenem and azithromycin, in addition to the vancomycin. Blood cultures drawn on admission showed no growth to date. Due to worsening clinical status, repeat blood cultures were sent, as well as sputum culture, respiratory pathogen panel, cytomegalovirus (CMV) and herpes simplex virus 1 and 2 antibodies (HSV1, HSV2). Buccal mucosa biopsy was deferred for several days pending stabilization of the patient’s respiratory status. A transthoracic echocardiogram was obtained to assess cardiac function in the setting of hypoxia and revealed an ejection fraction of 65% and moderate pericardial effusion without cardiac tamponade physiology. A CT chest without contrast revealed a multifocal pneumonia and a spiculated nodule in the right middle lobe (Figure 3). 

On hospital day seven, the result of an esophagogastroduodenoscopy conducted for dysphagia evaluation at the outside hospital prior to transfer was obtained. The report mentioned a non-bleeding esophageal ulcer and biopsy of the lesion was positive for cytomegalovirus inclusions with a negative HSV1 stain. Later, both CMV immunoglobulin M (IgM) and immunoglobulin G (IgG) serologies came back positive with a CMV Quantitative DNA polymerase chain reaction (PCR) of 4,549 IU/mL (7824.28 copies/mL). HIV screen was negative. 

**Figure 4:**
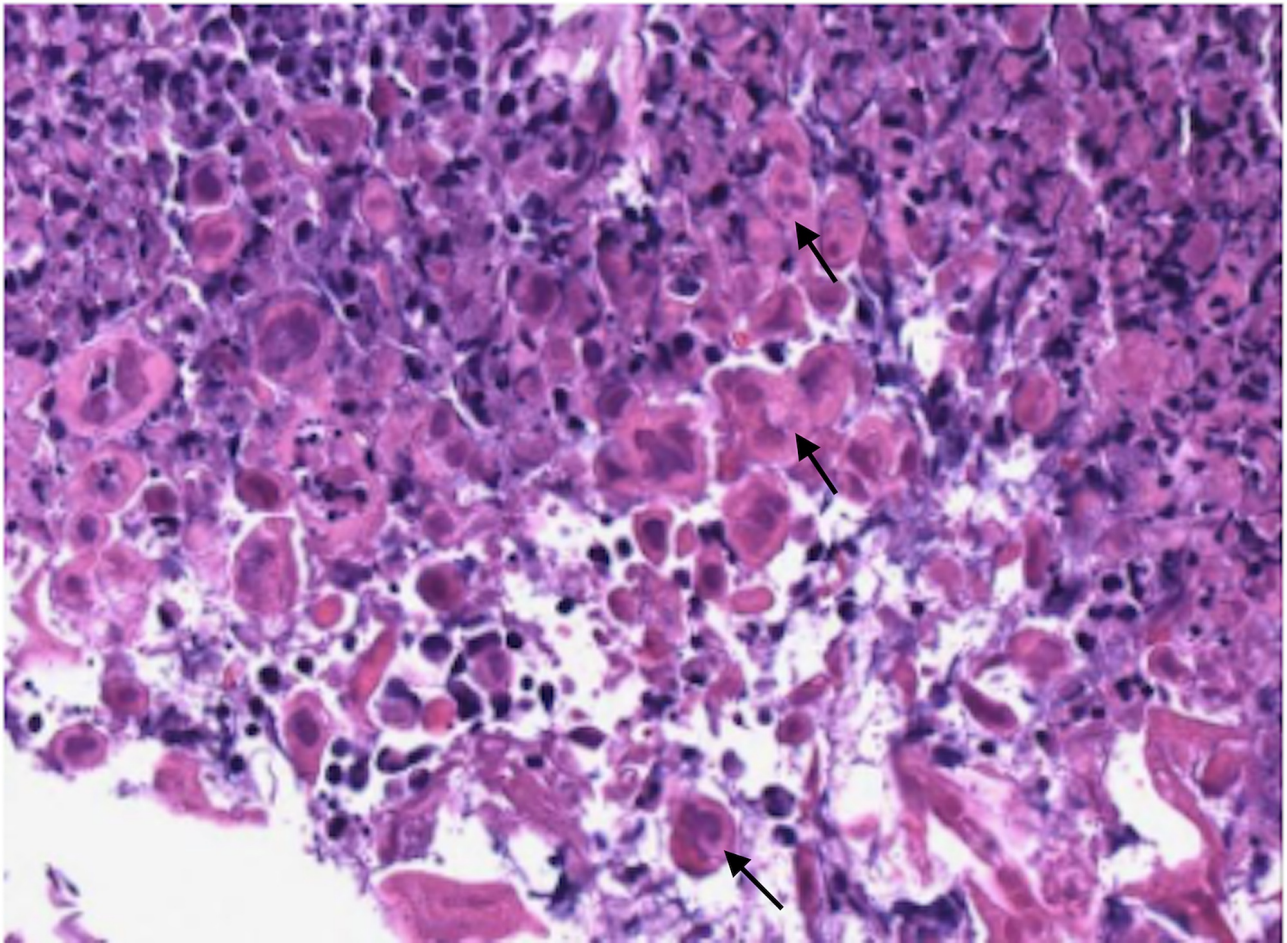
H&E Stain of Buccal Lesion Specimen Demonstrating HSV Infection Hematoxylin and eosin (H&E) stain of the left buccal lesion biopsy sample showed typical histology features of herpes simplex virus (HSV) infection, including the molding of nuclei, margination of chromatin, and multinucleation (white arrows).

**Figure 5:**
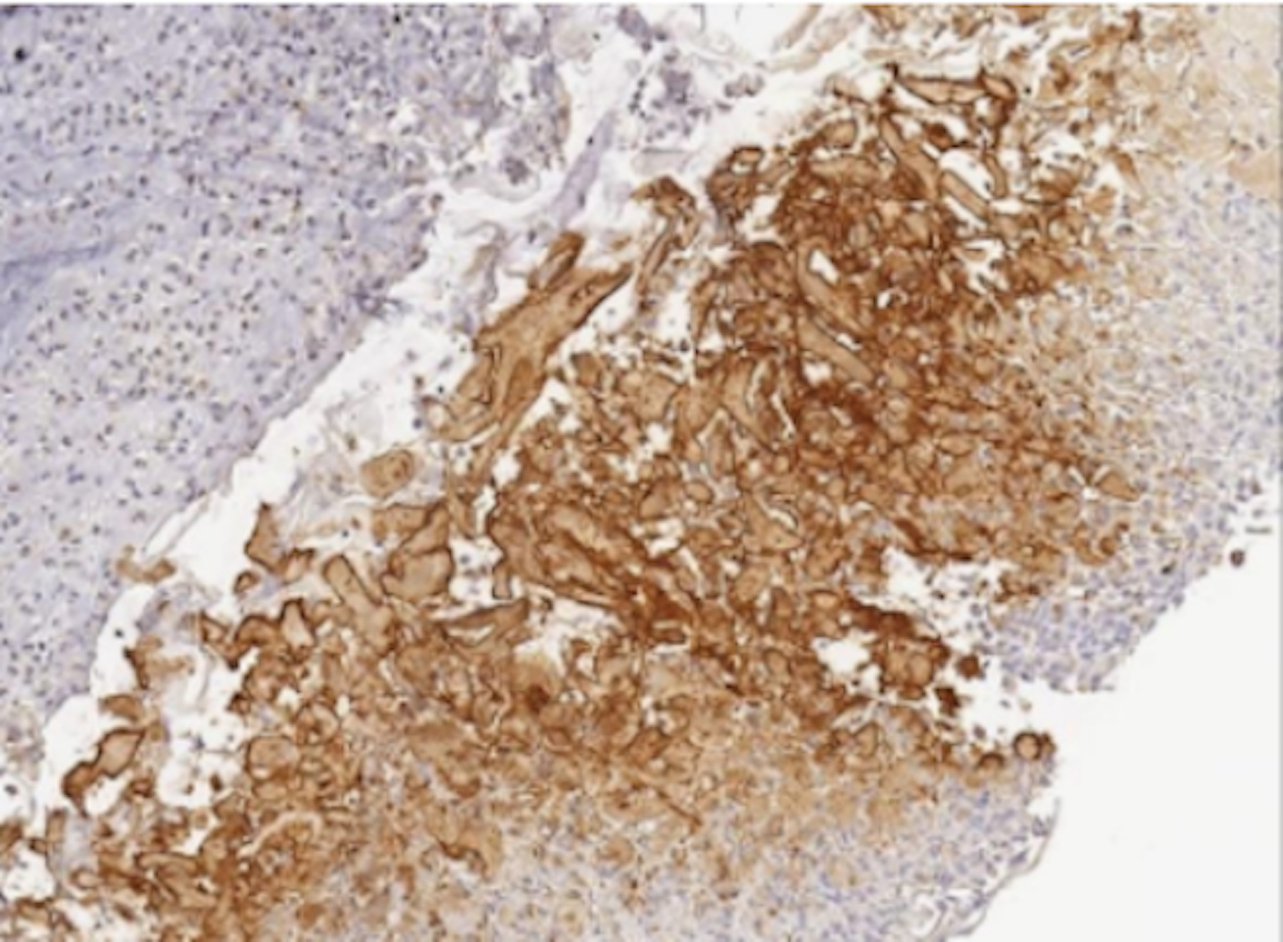
HSV-Specific Histologic Stain of Buccal Mucosal Sample Immunohistochemistry stain of left buccal mucosa specimen specific for herpes simplex virus (HSV) 1 & 2 was also positive.

Vancomycin and azithromycin were discontinued. The patient was started on ganciclovir and continued on meropenem. Subsequently, his respiratory status began to improve. He was weaned off of BiPAP and high flow oxygen, saturating at 95 to 100% on 6L nasal cannula that continued to be titrated down. On day 11 of admission, ENT performed a bedside biopsy of the left buccal lesion and GI placed a PEG tube for nutrition. The intravenous ganciclovir was switched to valganciclovir to be administered via the PEG tube. Final pathology of the buccal ulcer revealed intracellular features consistent with herpes simplex virus and was positive for HSV1 and HSV2 immuno-stains (Figures 4, 5).

**Figure 6:**
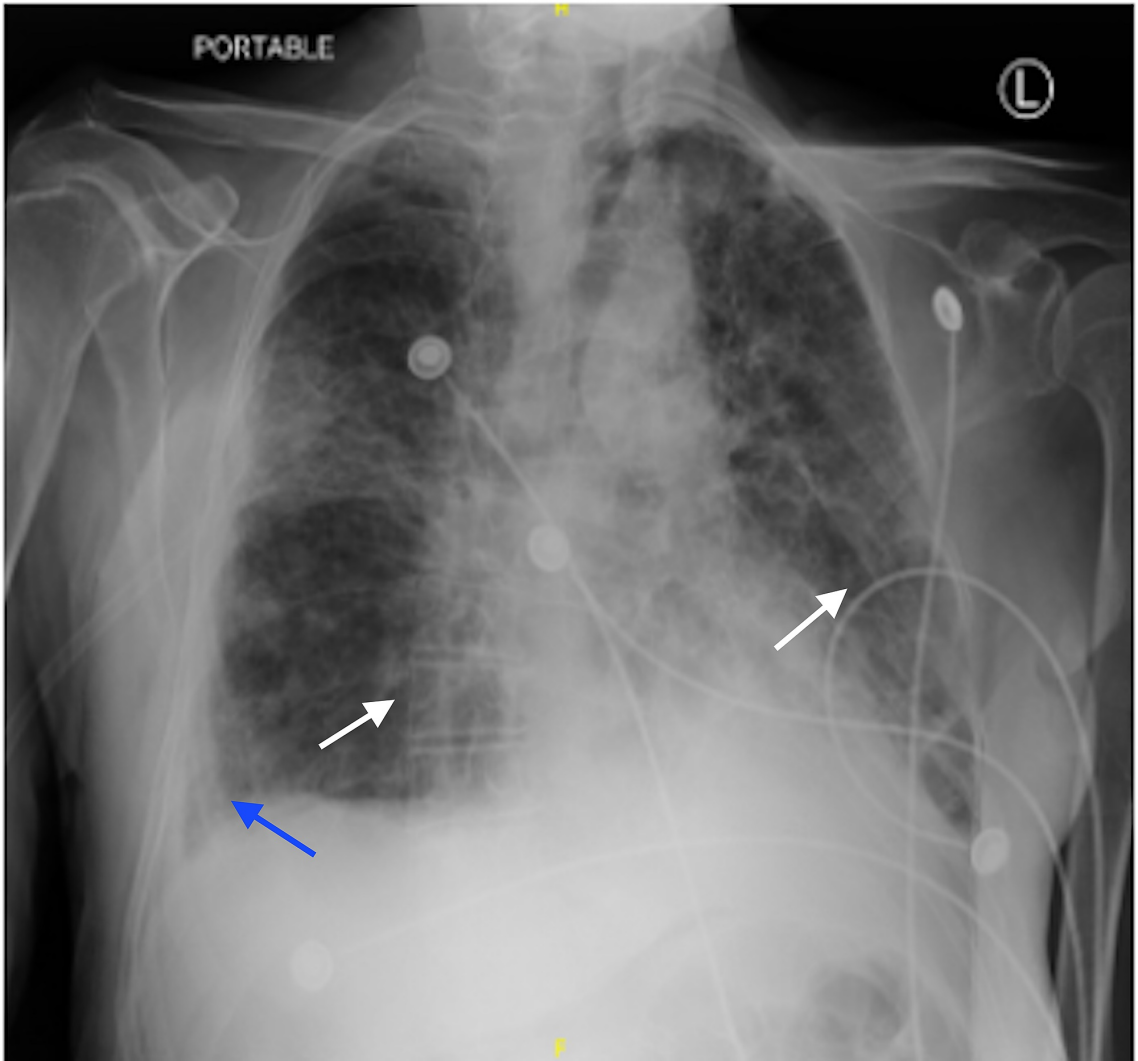
Repeat Chest X-ray after Antiviral Therapy Initiation Chest X-Ray taken on day four of antiviral therapy. Bilateral consolidations decreased compared to image B (white arrows). Pleural effusion is also decreased compared to image B (blue arrow).

Meropenem was discontinued after a 10-day course and he was switched from valganciclovir to intravenous valacyclovir on hospital day 15. The patient’s white blood cell count remained within normal limits and blood cultures showed no growth throughout the duration of the admission. Repeat chest x-ray after four days of antiviral medication demonstrated improvement of the bilateral consolidations (Figure 6). On hospital day 16, the patient was deemed stable for transfer back to the outside hospital for continued care. 

The patient was transferred back to the outside hospital upon stabilization of his respiratory status and completion of the buccal ulcer biopsy for continuation of care. The patient and his family were unable to be reached for one-month and six-month follow-ups after the transfer. As a result, an update on the patient’s clinical course after transfer could not be obtained.

## Discussion

Cytomegalovirus infection has been well-documented in immunocompromised patients, specifically in transplant patients on immunosuppressive therapy, patients on long-term dialysis or patients infected with HIV [1]. In these patients, the pathogenesis of CMV infection involves either reactivation of the latent virus or failure to clear a primary CMV infection due to the host’s weakened immune response [1]. According to a systematic review published in 2008, there are very few case reports of CMV infection in immunocompetent patients as the clinical course is often benign and self-limiting [2]. We presumed our patient was immunocompromised due to a recent course of high-dose systemic steroid therapy or a potential underlying malignancy as suggested by the presence of a spiculated lung nodule on CT chest. This nodule could represent recurrent or new malignancy given the patient’s history of oropharyngeal squamous cell carcinoma, extensive prior tobacco use, and age. Regardless of immune status, fulminant CMV infection in patients without HIV, such as our patient, was thought to be relatively rare until recently. One systematic review identified 290 patients without HIV that developed severe CMV infection. Among these patients, the most common sites of disseminated CMV infection involved the colon and central nervous system [2]. However, CMV infection involving other parts of the gastrointestinal tract and the respiratory system has not been well described in the literature. 

In our patient, disseminated CMV infection affecting the gastrointestinal tract (esophagitis) and respiratory system (pneumonitis) was a diagnosis of exclusion (later confirmed serologically) as his fever and worsening respiratory status with increased oxygen requirement persisted despite several days of broad-spectrum antibiotic therapy and negative blood cultures. It is also possible that the CMV infection contributed to our patient’s pericardial effusion as one case describes pericardial effusion and serositis secondary to CMV in an otherwise healthy patient [5]. The detection of CMV-IgM along with the endoscopic biopsy results confirmed our suspicion for disseminated CMV infection. Treatment with ganciclovir was initiated, after which our patient clinically improved. Several studies showed that only a few doses of antiviral medications are required to influence the course of disease [6,7]. Early recognition and initiation of ganciclovir in our patient successfully reduced the patient’s oxygen requirements and ultimately avoided intubation and intensive care unit (ICU) level of care. 

When the patient was finally stable enough to undergo ENT biopsy of his buccal ulcer, we were surprised when the final pathology revealed herpes simplex virus and not CMV. CMV esophagitis with concurrent HSV oral ulcer has been described in several reports in patients with HIV and AIDS [8-10]. Yet, this co-infection with CMV and HSV is exceedingly uncommon in the non-HIV infected population. There are two case reports published of CMV and HSV co-infection in patients without HIV. Both patients described received high dose steroid therapy prior to simultaneous viral infection, similar to our patient [3,4]. In each case, steroid therapy resulted in severe bone marrow suppression, leukopenia and subsequent synchronous infection. Immunosenescence, the age-related deterioration of the immune system, may have also predisposed our patient to concurrent CMV and HSV infections. CMV infection most commonly occurs in childhood, but the immunocompetent host is often asymptomatic [11]. Studies have shown that over time, CMV infection results in oligoclonal proliferation of CMV-targeting CD8+ T-cells, which may in turn reduce the T-cell response to other pathogens, including other viruses within the herpesvirus family [12]. In this way, a blunted immune response secondary to CMV-related immune dysregulation, and therefore contribution to immunosenescence, could have prompted the reactivation of HSV in our patient. 

It is interesting that our patient’s odynophagia was due to CMV and HSV found in two separate ulcers within the gastrointestinal tract (the esophageal ulcer was CMV positive while the buccal ulcer was HSV1 and HSV2 positive). The HSV immunohistochemical stain is specific to HSV glycoproteins, and while it cannot distinguish between HSV1 and HSV2 infection, the stain does not cross-react with other viruses, such as CMV [13]. Therefore, it is unlikely that the sample falsely identified HSV and not CMV in the buccal ulcer. The above cases of CMV and HSV co-infection in patients without HIV identified the two viruses within the same ulcer, which increased the risk of hemorrhage and perforation at the site of the ulcer [4]. The risks and prognosis associated with multiple viral infections at different sites along the gastrointestinal tract remain unclear. 

## Conclusions

This case demonstrates the importance of considering cytomegalovirus infection in the non-HIV infected patient with persistent fever, respiratory distress, or dysphagia despite antibiotic therapy, particularly in the setting of recent prolonged or high-dose steroid use. CMV and HSV infection can occur simultaneously at distinct sites. In fact, the long term effects of CMV infection may contribute to immunosenescence and therefore predispose patients to the reactivation of other herpesviruses, such as HSV. The overall prognosis associated with multiple viral infections at different sites along the gastrointestinal tract remains unclear and further studies would assist in elucidating this. Regardless, early identification and initiation of antiviral therapy can positively impact the hospital course, reduce the need for ICU level of care, and ultimately prevent poor outcomes. 
